# Simplifying Electrode Design for Lithium-Ion Rechargeable Cells

**DOI:** 10.1021/acsomega.2c04966

**Published:** 2022-10-11

**Authors:** Tianye Zheng, Steven T. Boles

**Affiliations:** † Department of Electrical Engineering, The Hong Kong Polytechnic University, Hung Hom, Kowloon 999077, Hong Kong; ‡ Department of Energy and Process Engineering, Norwegian University of Science and Technology, Høgskoleringen 1, 7491 Trondheim, Norway

## Abstract

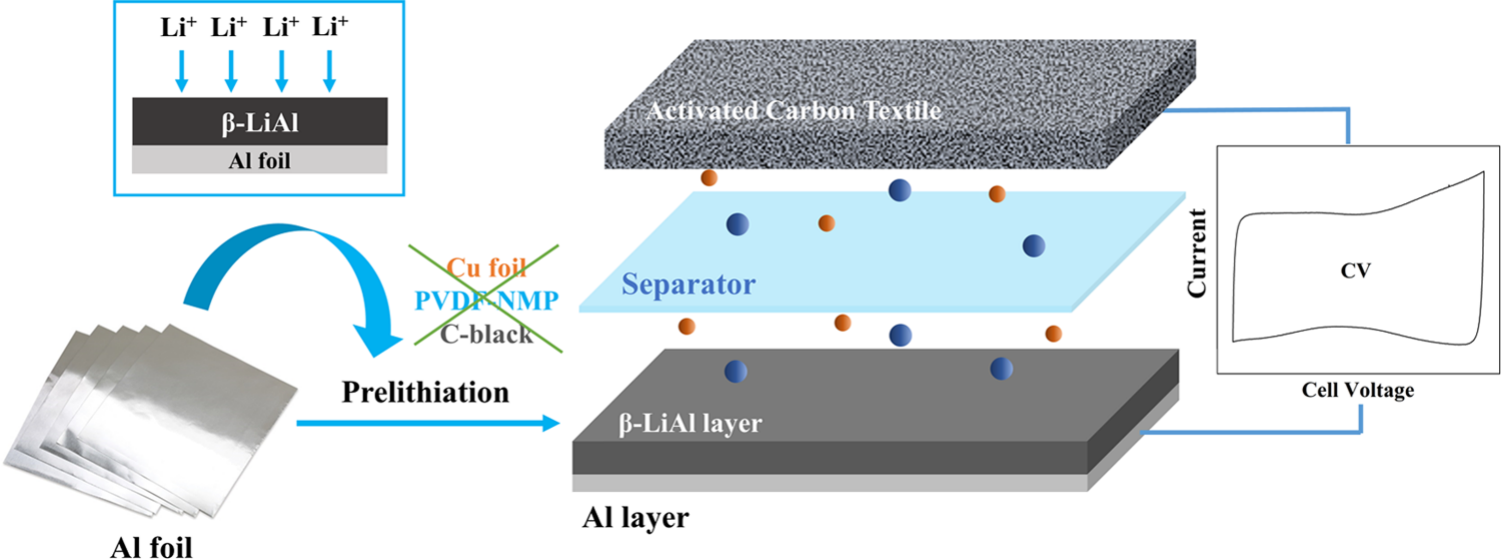

In the race to increase lithium-ion cell manufacturing, labor and energy costs quickly ascend to become chief concerns for building new facilities, as conventional electrode designs need significant resources during fabrication. Complicating this issue is an empirical trade-off between environmental friendliness and ethical sourcing. To circumvent this paradox, modified cell designs that employ foils and textiles can significantly change manufacturing considerations if their simple construction can be matched with competitive performance. In this work, we demonstrate one possible cell design for a lithium-ion device that utilizes a fabric and a foil for the cathode and the anode, respectively. For the anode, a prelithiated aluminum foil is chosen, as the room-temperature solubility range of the LiAl phase is well-suited to uptake and release lithium, all while reducing energy or cost-intensive production steps. The cathode is composed of activated carbon fiber textiles, which offer a scalable path to realize sustainability. With such benefits, this device design can potentially change the calculus for the mass production of energy storage devices.

## 1 Introduction

**Figure 1:**
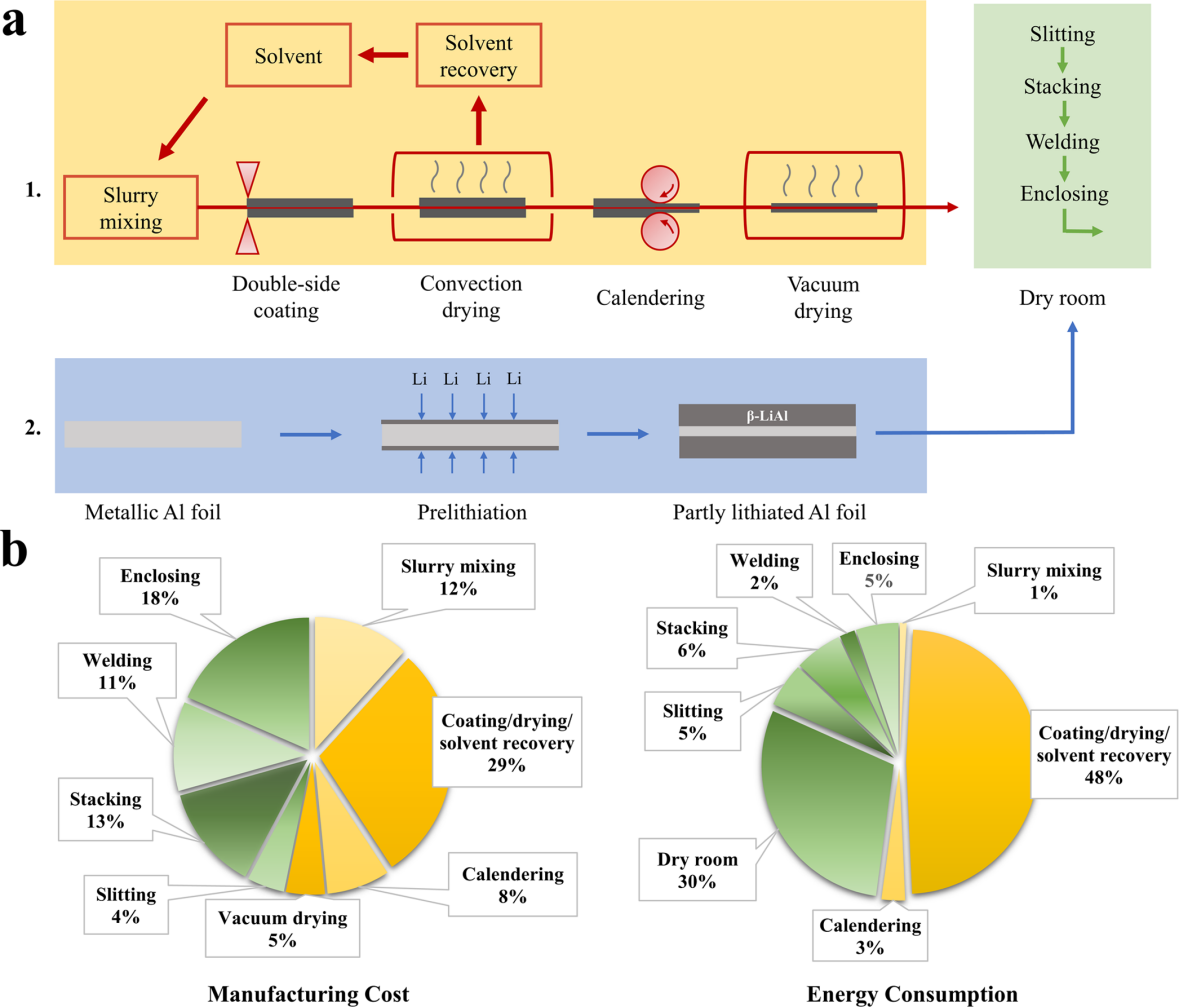
(a) Schematic of electrode fabrication for lithium-ion cells. Pathway 1: conventional composited-based manufacturing (yellow background). Pathway 2: the solid electrode fabrication proposed in this work (blue background) that aims at replacing the powder and slurry steps. (b) Breakdown of manufacturing cost and energy consumption for Li-ion cell production until the enclosing step. The data are extracted from Liu et al., where the labor cost is not considered.^2^

The widespread adoption of electric vehicles is supposed to help the planet, but does massive battery production create as many problems as it solves? Conventional electrode manufacturing for lithium-ion rechargeable cells, such as batteries, supercapacitors, and other hybrid devices, is composite-based, consisting of slurry mixing, coating, calendering, and vacuum drying (Pathway 1; Figure 1a). Both manufacturing costs and energy consumption associated with this cell design are concerning (Figure 1b),^1,2^ with the composite structure necessitating significant engineering and optimization that is unique to every manufacturer. In addition, these processes historically involved various chemicals that complicate occupational safety (e.g., *N*-methyl-pyrrolidone, NMP), but even if these risks are mitigated, overriding sourcing issues from elaborate material mixes create supply chain and logistical considerations, which remain challenging to eliminate.

In this work, we aim to demonstrate a simplified electrode design that can potentially be a more sustainable alternative to conventional, resource-intensive electrode fabrication. By prelithiating an aluminum metal foil (Pathway 2, Figure 1a), not only are energy-intensive heating and drying processes no longer necessary but the use of a copper foil (i.e., current collector) is also completely excluded from the manufacturing of the anode.^3^ This eliminates the powder–slurry steps prior to dry room processing and replaces them with single prelithiation, which can be done either (electro-)chemically or mechanically, perhaps even as reel-to-reel at a full scale. Although it is debatable to what extent a moisture-controlled environment (e.g., a dry room) is necessary for the prelithiation process (blue background in Figure 1a), it has been demonstrated elsewhere that LiAl could be quite stable in ambient air by artificially forming a solid electrolyte interface (SEI) layer^4^ or via shot peening treatment of Al foils before lithiation.^5^

While the dual functionality of an Al foil anode serving as both an electronic conductor and a lithium host represents a significant advancement for lithium-ion cell designs, the quest for a foil-based cathode may be more problematic, since the use of ceramic oxides is largely unavoidable for high energy density devices. Indeed, a composite with a malleable metal matrix may be highly effective on the cathode side for realizing the construction of solvent- and binder-free full cells. Innovations like semisolid electrode processing^6^ or hot rolling^7^ may help with several production aspects, but the electrode remains composite-based without substantially changing the architecture. On the other hand, textiles may offer an immediate demonstration for cathodes in hybrid lithium-ion capacitors. Specifically, activated carbon fabric (ACF) cathodes are commercially available and instantly satisfy the requirements for a rechargeable device that is capable of competitive figures of merit, at least for a hybrid capacitor. With solid LiAl on Al design proposed in this study, issues such as environmental impact, occupational health/safety, and global supply chain logistics, which are associated with conventional electrode fabrication, can potentially be lessened during cell manufacturing.^2,8^

From the perspective of a product life cycle, the composite nature of conventional electrodes also makes lithium-ion cells problematic.^9^ With recycling efforts reliant on pyrometallurgy, organic components, such as electrolytes and binders, must all be vaporized, and further hydrometallurgical processes necessitate strong oxidants (e.g., H_2_SO_4_/H_2_O_2_) to treat the shredded or granular residual pieces of the battery. Using today’s methods, only a few valuable transition metals (e.g., Ni, Co, etc.) are being recovered due to financial and technical challenges.^10^ So although this recycling process is established for general consumer cells, numerous drawbacks, such as the release of toxic gases, high energy consumption for heating, and low selectivity, should not be neglected.^11^

## 2 Results and Discussion

### 2.1 Solid Anode Fabrication

**Figure 2:**
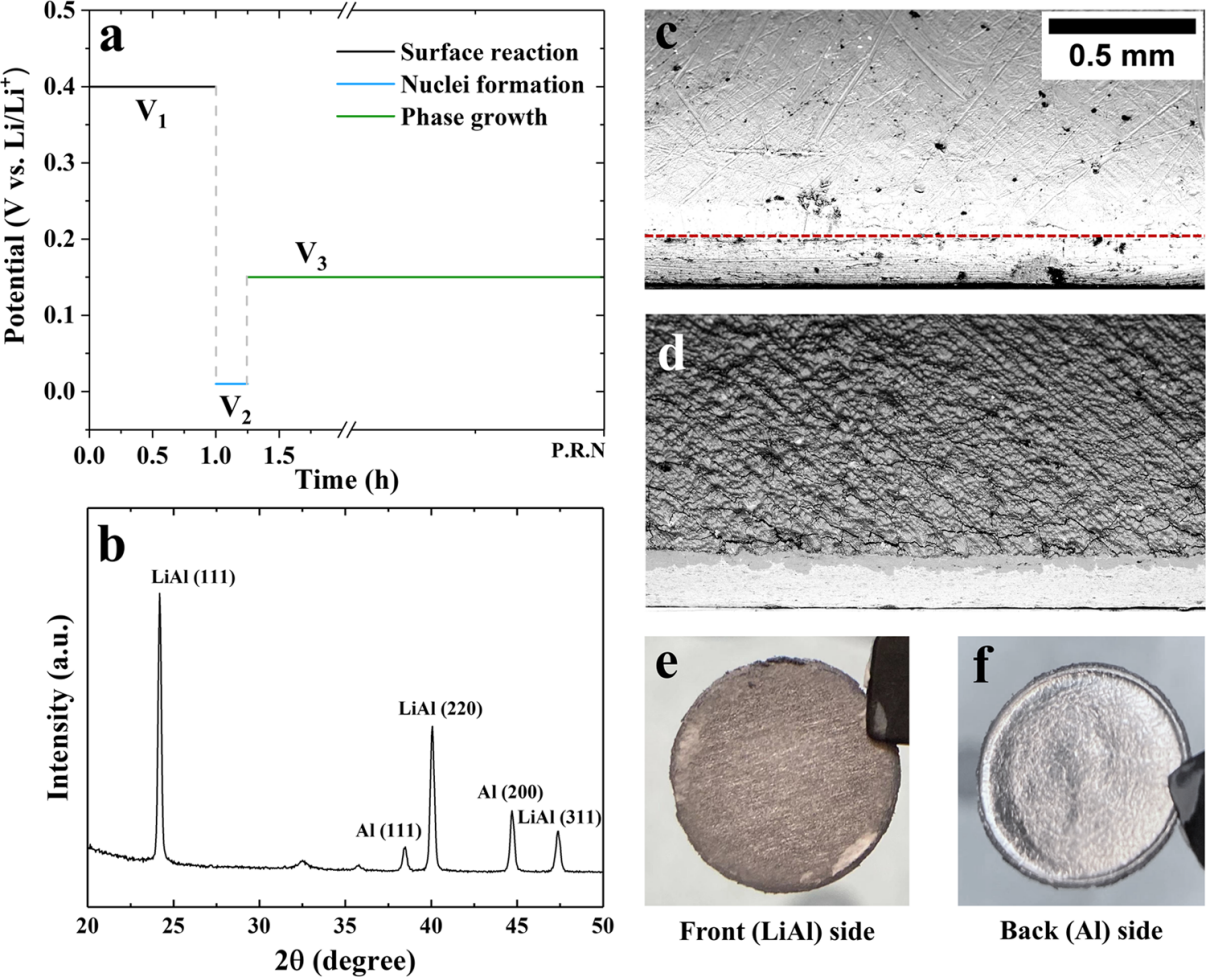
(a) Electrochemical protocol for preparation of β-LiAl on the Al electrode. (b) The X-ray diffractogram obtained from the front side of the prepared electrode (β-LiAl). SEM images taken using a 90° sample holder with a tilting angle of 45° for (c) a pristine Al foil (the red dashed line separates the surface and the cross section) and (d) a partially lithiated Al foil with a homogeneous β-LiAl layer covering its surface, following the protocol presented in (a). Macroscopic views of the prepared electrode: (e) front side and (f) back side.

The key enabler in this demonstration is the solid anode, prepared by partly lithiating an aluminum metal foil. As presented in Figure 2a, the electrochemical protocol is developed based on nucleation theories and lithiation kinetics.^12^ The surface reaction step (V_1_) aims to largely isolate the irreversible lithiation process. After holding the Li/Al half-cell at 0.40 V versus Li/Li^+^ for an hour, following the strategy described by Geronov et al.,^13^ the potential is adjusted to a level as low as 0.01 V (V_2_) to form a great amount of β-LiAl nuclei on the Al surface within only 15 min (Figure S1). When the potential jumps back to a moderate level at 0.15 V versus Li/Li^+^ (V_3_), the subsequent phase transformation will mostly take place at the positions where the β-LiAl nuclei already exist, resulting in a layer of β-LiAl that homogeneously covers the Al foil surface with a targeted thickness based on anticipated device capacity.^14^

The prepared electrode yields distinct peaks of crystalline β-LiAl and Al in the X-ray diffractogram (Figure 2b), while the bilayer nature of the prepared electrode is revealed by the scanning electron microscopy (SEM) images (Figure 2c,d). From a macroscopic view (Figure 2e,f), the lithiated surface exhibits a grayish color, while the back side of a single-side electrode remains the silvery white of aluminum metal. For comparison, another Al foil was prelithiated by directly holding the potential at V_3_. One can find from the SEM image in Figure S2 that the distribution of β-LiAl becomes discrete and inhomogenous, highlighting the necessity of the electrochemical protocol presented above. Besides the electrochemical prelithiation, it has been shown elsewhere that simple mechanical rolling or chemical lithiation may also be similarly effective, although confirmation of the product composition is still needed.^15^

### 2.2 Device Performance Assessment

**Figure 3:**
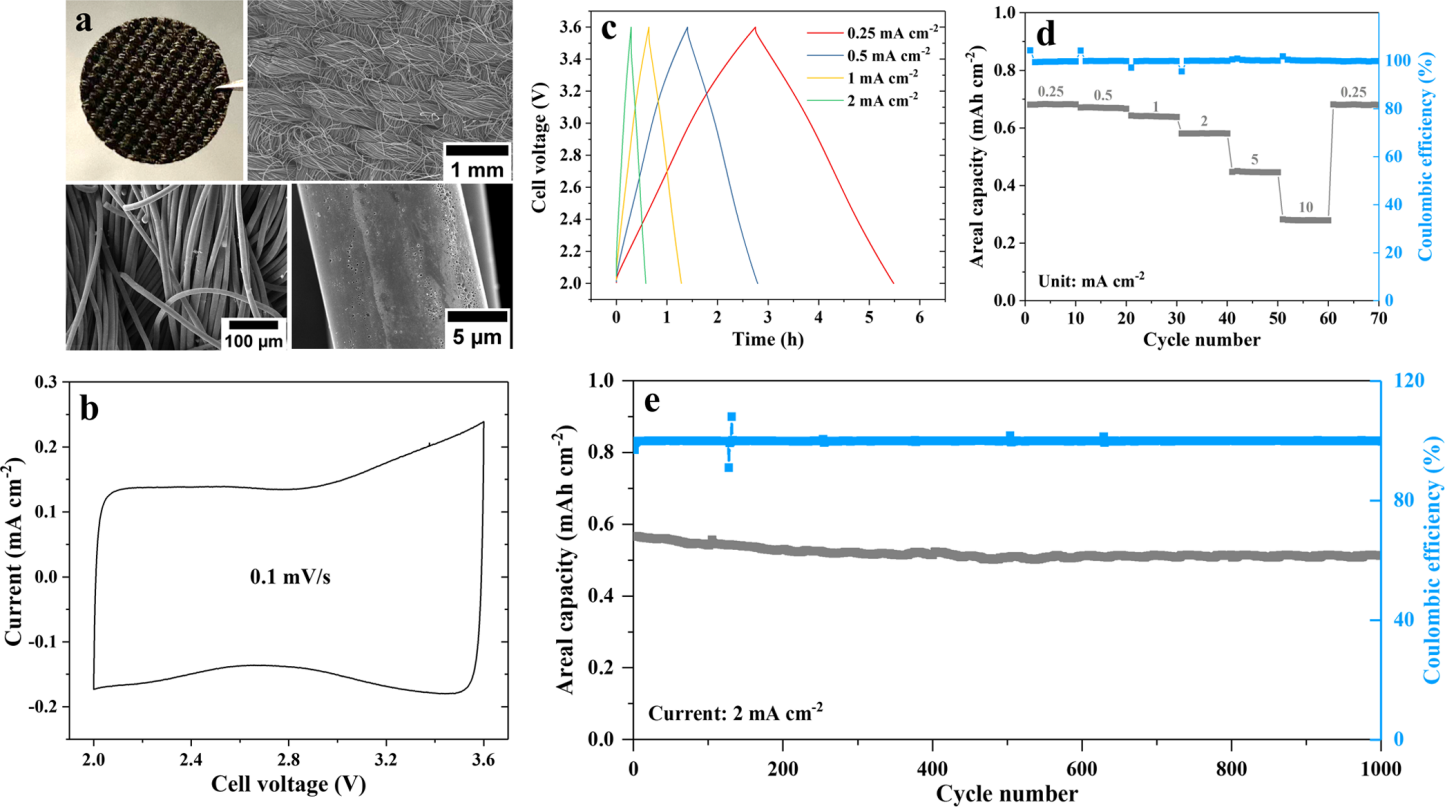
(a) Structure and morphology of the self-standing activated carbon fabric (ACF) including photographic and SEM images; (b) typical cyclic voltammogram, (c) galvanostatic profiles at various C rates, (d) the rate capability evaluation, and (e) the cycling performance obtained from the full cell consisting of β-LiAl on the Al foil anode and the ACF cathode.

To assess the feasibility of having a rechargeable cell with solid electrodes, a hybrid lithium-ion capacitor has been assembled using β-LiAl on the Al foil (0.1 mm thick before prelithiation) and the self-standing ACF (ca. 0.4 mm thick; Kuraray, Japan) as the anode and the cathode, respectively. The scanning electron microscopy (SEM) images of ACF in Figure 3a show a woven structure, of which a single fiber is roughly 15 ± 5 μm in diameter. Numerous pores (∼2.2 nm) can be observed on the fiber surface under SEM, indicating the huge surface area (∼1400 m^2^ g^–1^, obtained from a Brunauer–Emmett–Teller (BET) test). Based on fiber geometry, it is quickly recognized that most of the carbon in the ACF serves as structural support, instead of providing sites for ion sorption during cycling. Hence, there remains a significant opportunity for increasing its specific capacity with surface-to-volume structural optimization.

Electrochemically, the cyclic voltammogram obtained from the lithium-ion capacitor exhibits a nearly ideal rectangular shape within a potential range between 2 and 3.6 V (Figure 3b). It is worth mentioning that a lithium metal foil is also paired with the ACF to compare the lithium storage at the anode side via lithium deposition/stripping to the cell of primary interest here, which utilizes the solubility range of β-LiAl. The cyclic voltammograms provided in Figure S3 show that the LiAl anode has better charge storage characteristics than the Li metal anode as low potentials are approached (i.e., a more rectangular curve), thus demonstrating some resistance to possible lithium plating at higher rates. Compared to the electrical double-layer capacitors (EDLCs) that are often charged from 0 V, the mid-point voltage is 2.8 V in this case, thereby resulting in a higher energy density (based on *E* = *C*_s_ × *V*_mid_). Correspondingly, the galvanostatic profiles (Figure 3c) also show an ideal symmetry between charge and discharge, indicating good charge efficiencies at various rates. As can be seen from Figure 3d, the rate capability of the lithium-ion capacitor has been assessed, delivering moderate areal capacities (i.e., lower than lithium-ion batteries (LIBs) but higher than electrostatic supercapacitors (SCs)). One may notice that ca. 85% of the capacity obtained at 0.25 mAh cm^–2^ is still possible to realize when the (dis)charging rate is increased to 2 mAh cm^–2^ (a factor of 8), resulting in a ∼6.8 times higher power density (8 × 85%) and indicating a superior rate capability within this current range.^16^ However, the capacity decreases dramatically by further increasing the rate. Therefore, the cycling performance is evaluated at 2 mA cm^–2^, where the energy and the power density are balanced, in line with application considerations, giving 1000 cycles with ca. 90% capacity retention. Since the cycled capacity is limited by the ACF, the (de-)saturation of the β-LiAl layer should prevail instead of the α/β/α phase transformations.^14^ Compared to the poor cycling lives of Al anodes reported by others, our device takes advantage of the solubility range of β-LiAl that circumvents the intrinsic problems arising from the phase transformations, such as mechanical strain^17^ and formation of nanopores.^18^ The device delivers ∼1.6 mWh cm^–2^ at an areal power of ∼5.6 mW cm^–2^ (single-side), or a gravitational energy of ∼25.6 mWh g^–1^ at a gravitational power of ∼88.3 mW g^–1^, normalized to the total mass of both Al and ACF.

## 3 Perspectives and Outlook

At a first glance, the performance figures obtained from our device may not look as competitive as some of the best reported values in the literature. However, academic reports often present normalized energy/power densities that solely consider the mass of the active material, thereby unfairly comparing commercial products in well-known Ragone^19^ plots. For instance, the normalized values can be several orders of magnitude higher than those of commercial products but hold limited promise in real applications.^20^ Few reports take into account all of the cell components when reporting these values, so fair comparisons are essential to evaluate performance, and particularly so when electrode structures are similar.^21^

Many have recognized the chronic mismatch between academic reports and industrial metrics in various lithium-ion cells.^22^ Gogotsi and Simon suggested that a factor of 4 to 12 must be considered when extrapolating the energy/power densities from the material level to the device level due to the composite nature of electrodes.^23^ In this work, we estimate that this factor can potentially be reduced to ca. 2 by taking advantage of the solid electrode design that is free of any binder, conductive agent, and copper current collector (as detailed in Supporting Information).

**Figure 4:**
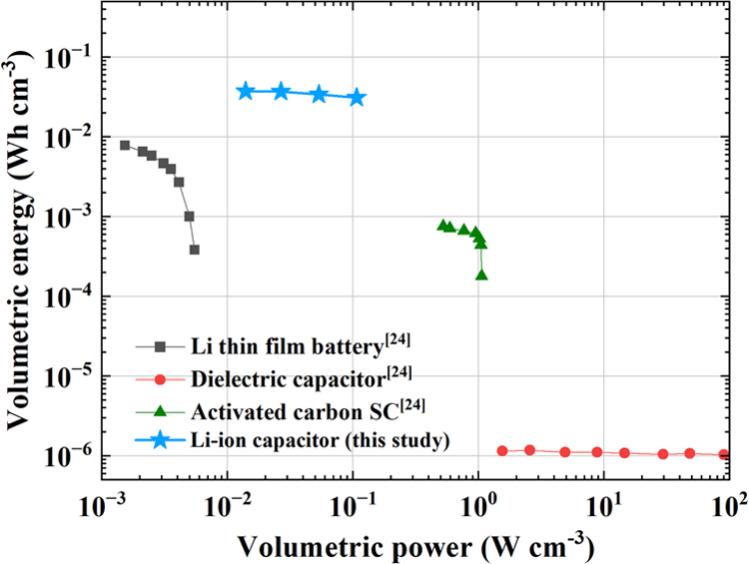
Ragone plot that compares the volumetric energy and power of the lithium-ion device in this study with that of other commercial energy storage devices, including a Li thin film battery, a dielectric capacitor, and an activated carbon supercapacitor (SC). These volumetric values are calculated based on the device stack, which includes the electrodes, the current collectors (if any), and the separator, i.e., packaging is not considered.

Moreover, the indicators of our lithium-ion capacitor are also incorporated into the existing Ragone plot to evaluate its performance. Since comparing with academic reports might be unfair due to the different denominators, we decided to include three types of commercial energy storage devices in Figure 4. Volumetric energy/power densities are used instead of gravitational ones, which may be misleading due to various uncertainties.^23^ It is worth mentioning that the values reported here are all normalized to the stack volume, which is defined by El-Kady et al. as the volume of the electrodes, the current collectors (if any), and the separator.^24^ As shown in Figure 4, our device sits somewhere between the upper left and the upper middle of the Ragone plot, indicative of high energy but moderate power density. The nature of our lithium-ion device can also be highlighted by the positions of other commercial devices in the same plot. For example, our device delivers volumetric energies that are roughly 2 orders of magnitude higher than that of the activated carbon SCs but at relatively lower power densities.

The electrochemical characteristics suggest that the lithium-ion capacitor might be suitable for applications aiming for a charge/discharge time between 10 and 60 min, which is slower than that of regular SCs (i.e., 10 s to 10 min) and faster than that of commercial LIBs (i.e., 1–3 h). Design-wise, commercial lithium-ion cells are made in different structures, including designs with a single pair of electrodes (e.g., flat-style, jellyroll) or with stacked electrodes (e.g., prismatic cells). Although Al foils may sacrifice some degree of flexibility depending on the thickness, the solid anode presented here may be readily available for flat or prismatic cell designs. Meanwhile, other device demonstrations with Al anodes, such as bipolar cell design,^25^ are also supporting the competitiveness of this technology.

Despite the pursuit of Al as an anode in lithium-ion rechargeable cells that has been explored for more than 55 years and commercialized for 30 years, breakthroughs have been missing to make these anodes competitive for state-of-the-art devices. We hope that an enriched understanding of the room-temperature solubility of β-LiAl opens up new opportunities for scaleable, high-performance lithium-ion energy storage devices. With the ability to fabricate the entire anode out of a single piece of Al foil, a simplified manufacturing process improves the prospects for sustainability across multiple dimensions. In addition to the carbon fabric cathode used in this report, solid foil ceramic–metal composites can be readily envisioned to offer similar device benefits in full-cell lithium-ion batteries. Importantly, the reduced material complexity of foil and fabric cells has the potential to make disassembly less challenging and can push lithium-ion technologies toward a more circular economy with benefits for generations to come.

## 4 Experimental Section

### 4.1 Aluminum Foils

The Al foil electrodes used in this study were obtained from Alfa Aesar. The thicker Al foil (0.25 mm, ø = 12 mm, 99.9995%) was selected for conducting SEM experiments, such that the cross-sectional views can be revealed more easily. The thinner Al foil (0.1 mm, ø = 12 mm, 99.997%), which mimics the commercial electrode thickness, was used in the full cell assessment.

### 4.2 Electrochemical Processes

All electrochemical procedures/tests of conventional coin-type cells were performed using a VMP potentiostat (Biologic Technologies, France), including prelithiation, cyclic voltammetry, rate tests, and cycling assessment. The electrochemical prelithiation was performed in a half-cell via the following multistep process: (1) the Al foil electrode was first held at 0.4 V versus Li/Li^+^ (V_1_) for 1 h to minimize the charge contributed by the surface reaction at higher potentials; (2) the potential was then set to a very low level at 0.01 V versus Li/Li^+^ (V_2_) to form a large amount of β-LiAl nuclei within a short period of time (i.e., 15 min);^13^ (3) lastly, the potential jumped to a moderate level at 0.15 V versus Li/Li^+^ (V_3_) to facilitate the following phase transformation until 50 μm lithiation depth was achieved, which referred to 0.005 cm × 1 cm^2^ × 2.7 g cm^–3^ × ∼1000 mAh g^–1^ = ∼13.5 mAh cm^–2^.

### 4.3 Scanning Electron Microscopy (SEM)

Swagelok cells that can be easily disassembled were used here. When the Al foil was lithiated/delithiated to the desired state of charge/discharge, the Swagelok cell was disassembled in an argon-filled glovebox. The partly lithiated/delithiated Al foils underwent a series of grinding processes using sandpapers from #1000 to #5000 to create a flat and smooth cross section. A specifically designed transfer system (Leica VCT100) allowed for immediate sample transfer from the glovebox to the SEM system (Zeiss Merlin) without exposure to air. SEM images were acquired at an acceleration voltage of 6 kV, using a backscattered electron (BSE) detector, such that the β phase distribution can be revealed.

### 4.4 Full Cell Assembly

A full cell device was assembled with self-standing activated carbon fibers (ACF; 0.4 mm, ø = 12 mm) provided by Kuraray Co., Ltd, Japan, and a 0.1 mm thick Al foil that was prelithiated to 50 μm. The separator was a monolayer microporous membrane (25 μm thick, Celgard 2400). Various current densities were used to assess the cell performance with a voltage window between 2 and 3.6 V. The electrolyte used for all cases was LP57 (LiPF_6_ in EC/EMC 3:7).

### 4.5 Calculations of Volumetric Energy and Power

The cell energy (*E*) was calculated based on the equation *E* = *C* × *V*_mid_, where *C* and *V*_mid_ are the capacity and the mid-point voltage of the lithium-ion device, respectively. The cell power can be obtained by dividing the cell energy by the discharge time. The volumetric energy and power were then calculated by normalizing to the stack volume, which summed up the thicknesses of the ACF, the Al foil, and the separator: 0.4 mm + 0.1 mm + 0.025 mm = 0.525 mm cm^–2^.

## Supporting Information Available

Scanning electron microscopic images of lithiated Al electrodes during prelithiation steps; extrapolations of energy/power densities from a material level to a device level; and cyclic voltammograms of the LiAl vs ACF cell and the Li metal vs ACF cell (PDF)
