# Supplementary material for: Simplifying Electrode Design for Lithium-Ion Rechargeable Cells
Source: ACS Omega. 2022 Oct 11;7(42):37867–72. doi: 10.1021/acsomega.2c04966 (PMC9607682; doi:10.1021/acsomega.2c04966)
Supplement: Supplementary file 1 — ao2c04966_si_001.pdf [file ao2c04966_si_001.pdf]

Supporting Information

of

Simplifying Electrode Design for Lithium-ion Rechargeable Cells

*Tianye Zheng †, Steven T. Boles §,**

† Department of Electrical Engineering, The Hong Kong Polytechnic University, Hung Hom, Kowloon, Hong Kong

§ Department of Energy and Process Engineering, Norwegian University of Science and Technology, Høgskoleringen 1, 7491 Trondheim, Norway

*Corresponding author: steven.boles@ntnu.no

First author: darren.ty.zheng@connect.polyu.hk

Supplementary Figures

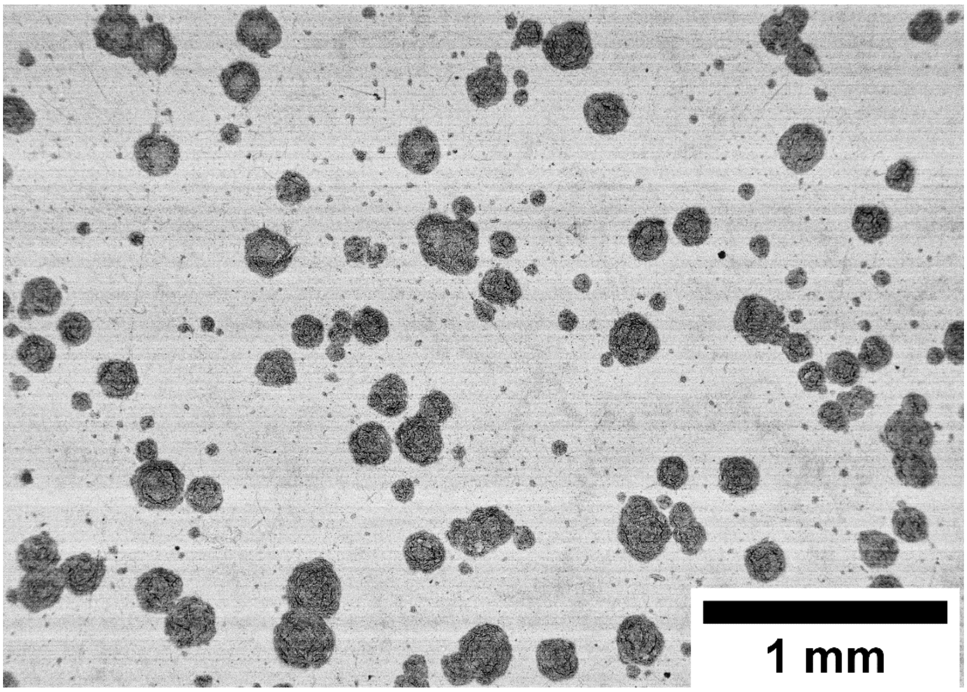

Figure S1. SEM image of the Al electrode taken from a Li/Al half cell that undergoes the first (V_1) and the second step (V_2) of the electrochemical protocol described in Figure 1 (end of the blue curve) in the manuscript.

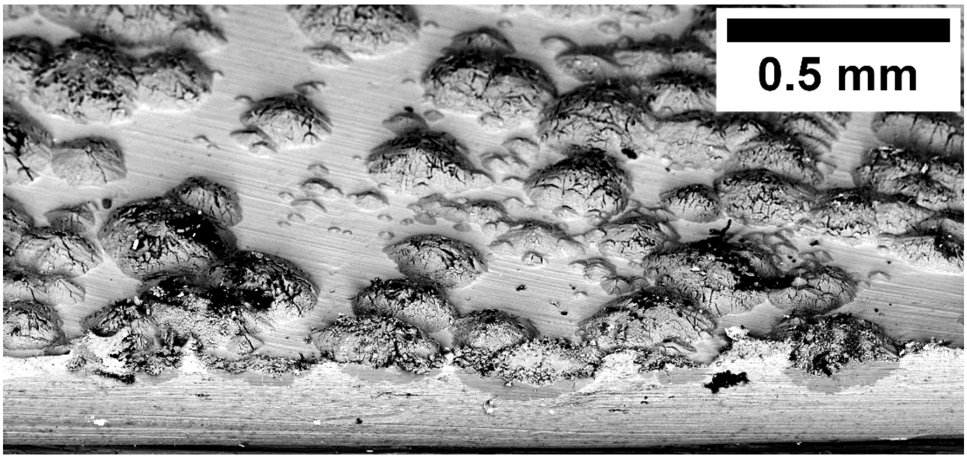

Figure S2. SEM image of the Al electrode taken from a Li/Al half cell that undergoes all the steps (V_1-V_3) of the electrochemical protocol described in Figure 1 (end of the green curve) in the manuscript.

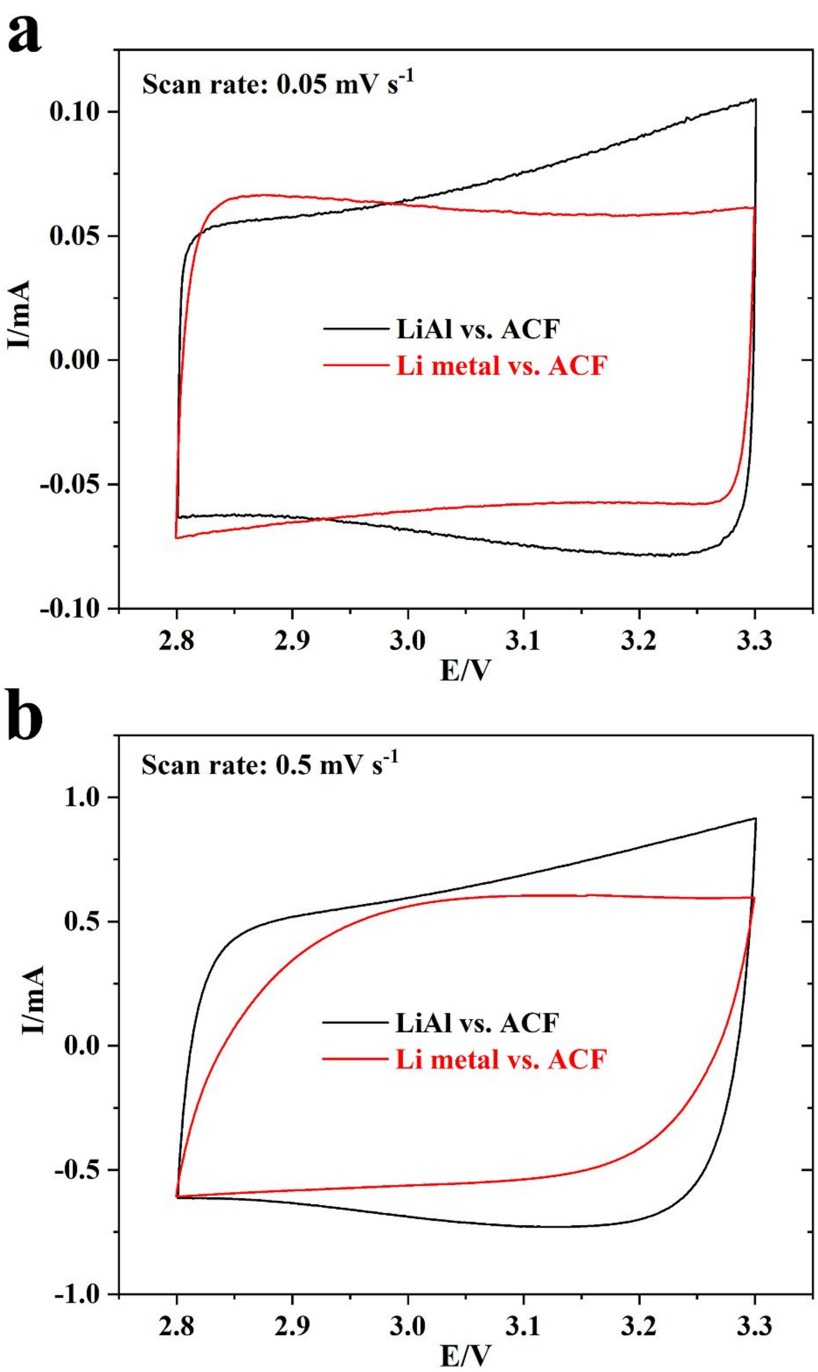

Figure S3. Comparisons of cyclic voltammograms between the LiAl vs. ACF and the Li-metal vs. ACF cell at (a) 0.05 mV s^{-1} and (b) 0.5 mV s^{-1}.

Extrapolation of energy/power densities

Gogotsi and Simon clearly stated in their perspective paper that a factor of 4 should be considered when extrapolating the performance from a material-level to a device-level in the energy storage field.[1] Although they focused on electrochemical capacitors, it is clearly stated that the considerations presented in their paper also apply to various lithium-ion cells. Critically, this factor of 4 is only valid for the electrodes that are comparable to the commercial ones. In other words, the electrode thickness or the active mass has to be beyond a certain value. An example of carbon-based supercapacitor electrodes is given: When the carbon dosage (i.e., active material) is decreased from 10 mg cm^{-2} (commercial standard) to 1 mg cm^{-2} (lab-scale), another three- to four-fold should be considered, giving an extrapolation factor of 12-16 instead of 4.

Supercapacitors. Using the above-mentioned standards of carbon-based supercapacitors, the electrochemically inactive components (including conductive carbon) are given by:

- Cu foil (8 μm thick): $8.96\ g\ cm^{-3} \times 1\ cm^2 \times 8\ \mu m = 7.2\ mg$
- Binder and carbon black (commercial standard): 5% weight percentage
- Binder and carbon black (lab-scale): 20% weight percentage

Consequently, the content of active material in a carbon electrode can be quantified as:

- Commercial cells: ${10\ mg}/{(0.53\ mg + 7.2\ mg + 10\ mg)} = 56\%$
- Laboratory cells: ${1\ mg}/{(0.25\ mg + 7.2\ mg + 1\ mg)} = 12\%$

Lithium-ion batteries. Considering the commercial standards of lithium-ion batteries, i.e., 1.5 to 2 mAh cm^{-2} single-side capacity,[2] the active material (e.g., graphite anode) per unit area would be:

- Graphite: $2\,mAh\,cm^{-2} \times 2\,sides/370\,mAh\,g^{-1} = 10.8\,mg\,cm^{-2}$

The calculated mass for a double-sided electrode is consistent with the standards claimed by Gototsi and Simon.[1] Binder and carbon black are then calculated using the same equations:

- Binder and carbon black (commercial standard; 5%): $10.8\,mg\,cm^{-2} \big/ (1-5\%)$ $-10.8\,mg\,cm^{-2} = 0.57\,mg$

Thus, the active material percentage in the conventional electrode design is:

- Commercial cells: $10.8\,mg \big/ (0.57\,mg + 7.2\,mg + 10.8\,mg) = 58\%$

In this manuscript, the reported performance metrics are normalized to the mass of the whole foil electrode. Therefore, the extrapolation factor of 4 mentioned above can hopefully be reduced to ~2 for the solid LiAl on Al electrode in the absence of the electrochemically inactive components that occupy roughly 42% of the total electrode weight.

REFERENCES

(1) Gogotsi, Y.; Simon, P. True Performance Metrics in Electrochemical Energy Storage. *Science* **2011**, *334* (6058), 917.
(2) Li, H. Practical Evaluation of Li-Ion Batteries. *Joule* **2019**, *3* (4), 911-914.
